# Case Report: Left bundle branch area pacing in cardiac resynchronization therapy increases conduction velocity

**DOI:** 10.3389/fcvm.2025.1620302

**Published:** 2025-10-03

**Authors:** Wenchang Zhang, Leyi Wang, Changjian He, Chunhua Ding

**Affiliations:** ^1^ Cardiac Department, Aerospace Center Hospital (Peking University Aerospace School of Clinical Medicine), Beijing, China; ^2^ Peking University Health Science Center, Beijing, China

**Keywords:** cardiac resynchronization therapy, left bundle branch area pacing, left bundle branch block, LBBB-induced cardiomyopathy, heart failure

## Abstract

Several studies have suggested that the application of left bundle branch area pacing (LBBAP) in cardiac resynchronization therapy (CRT) holds promise as a treatment modality for correcting left bundle branch block (LBBB) while concurrently enhancing left ventricular structure and function. However, it's noteworthy that current guidelines do not provide specific recommendations for the use of left bundle branch area pacing, underscoring the need for additional evidence regarding its safety and efficacy. In this context, we present a case report detailing the utilization of LBBAP-CRTD treatment in a patient with LBBB-induced cardiomyopathy. The investigation encompasses a thorough examination of the efficacy and safety of LBBAP-CRTD, with a particular emphasis on cardiac synchronization parameters. Written consent was obtained from the patient, and this case report adheres to the CARE guideline.

## Chief complaints and clinical findings

**Figure 1:**
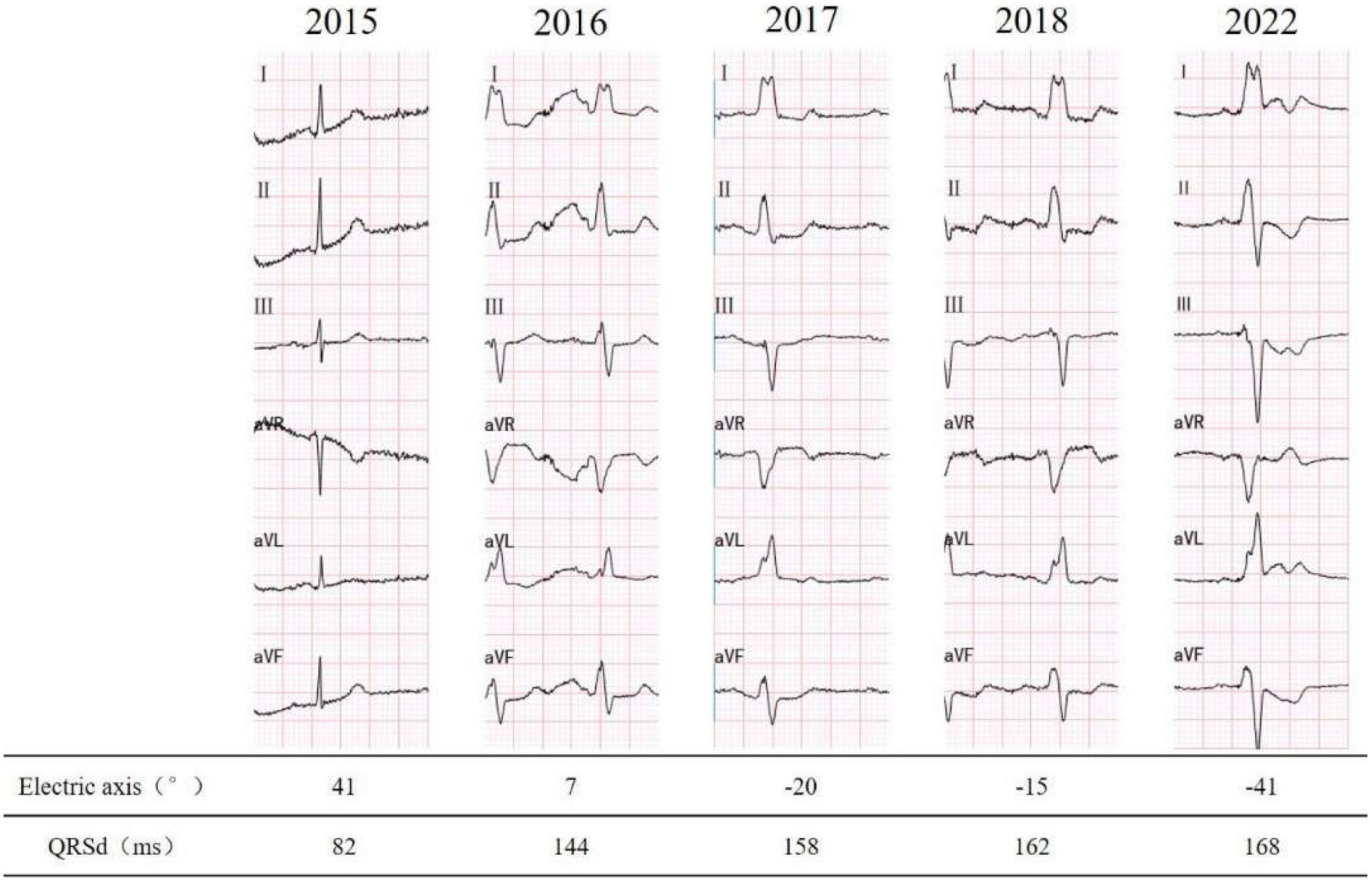
Past ECG records.

A 78-year-old male was admitted to the emergency department due to 4 h of unexplained, persistent palpitations and dizziness. There were no accompanying symptoms such as fever, cough, shortness of breath, chest pain, blurred vision, or syncope. The patient has been noted to have a left bundle branch block (LBBB) pattern on ECG since 2016 (Figure 1), with a more pronounced involvement of the left anterior fascicle. There is no history of hypertension, diabetes, atrial fibrillation, renal dysfunction, or thyroid disorders. The patient denies experiencing palpitations or chest discomfort during exercise or emotional stress, and reports good exercise capability. There is no family history of ischemic or structural heart disease.

The ECG revealed rapid atrial fibrillation with a ventricular rate of 135 bpm, coupled with a complete left bundle branch block (LBBB) pattern. The patient spontaneously restored sinus rhythm, leading to symptom alleviation. Baseline 12-lead ECG prior to left bundle branch area pacing (LBBAP), showing intrinsic rhythm with complete LBBB morphology. The QRS duration was approximately 168 ms, characterized by a wide, notched R wave in leads I, V5–V6, and a broad, deep S wave in V1, consistent with electrical dyssynchrony. A subsequent 24 h Holter monitoring was conducted, disclosing a transition to complete sinus rhythm, while maintaining a complete LBBB with left axis deviation.

Echocardiography shows left ventricular dilation with an ejection fraction of 32%, accompanied by impaired global contractility. The relevant parameters are outlined in Table 2.

**Table 2:** Parameters of LBBP-CRTD device at implantation and during follow-up.

Category	Parameter	At implantation	Follow-up		
			2 weeks	3 months	6 months
Battery (years)		9	10.1	10	9.8
VP (%)		99.9	99.9	99.9	99.9
RA lead	Amplitude (V)	0.5	0.5	0.5	0.5
	Pulse width (ms)	0.4	0.4	0.4	0.4
	Sensitivity (mV)	3.5	2.9	2.9	2.9
	Impedance (ohms)	399	399	399	399
LBB pacing lead	Amplitude (V)	0.5	0.75	0.75	1.25
	Pulse width (ms)	0.4	0.4	0.4	0.4
	Sensitivity (mV)	NA	NA	NA	NA
	Impedance (ohms)	551	551	551	551
RV lead	Amplitude (V)	0.75	0.75	0.5	0.5
	Pulse width (ms)	0.4	0.4	0.4	0.4
	Sensitivity (mV)	>20 mV	>20 mV	>20 mV	>20 mV
	Impedance (ohms)	532	532	532	532
Events	No event	No event	No event	No event	No event

Left ventricular mechanical synchronization parameters were assessed, and two-dimensional (2D) speckle tracking echocardiography (STE) disclosed notable intraventricular desynchrony in the left ventricle. The most prolonged delays were observed at the basal segments of the anterior and lateral wall. However, the interventricular mechanical delay (IVMD) was within the normal range.

The computed tomography (CT) scans of the head and chest revealed no notable findings. Subsequently, coronary angiography (CAG) was performed, uncovering a maximum of 30% stenosis in the mid right coronary artery (RCA) and 50% stenosis in the proximal left anterior descending artery (LAD). Importantly, both arteries exhibited TIMI grade 3 flow.

## Therapeutic interventions

**Figure 2:**
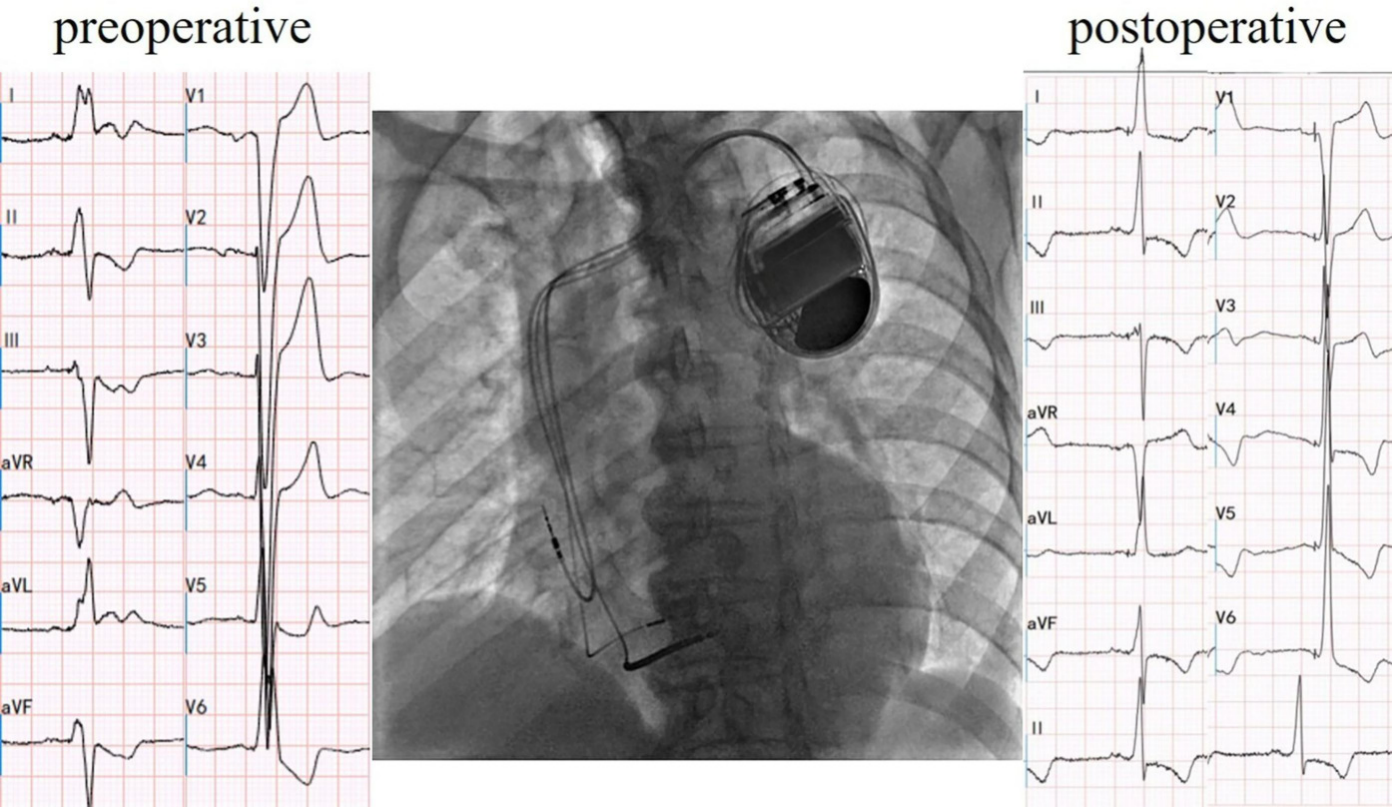
Cardiac resynchronization therapy-defibrillator with left bundle branch area pacing. Electric axis and QRS duration of postoperative were −6° and 106 ms, respectively.

Cardiac resynchronization therapy-defibrillator (CRT-D) with LBBAP was successfully implemented for the patient, achieving LBBAP with a QRS duration of 106 ms during the procedure(Figure 2). At the time of implantation, threshold testing revealed a transition from a wider, fused QRS to a narrower fully-paced QRS. The paced QRS showed a V6 RWPT of 93 ms and a V6–V1 interpeak interval of 10 ms. These findings are consistent with LBBAP. Notably, left axis deviation was not observed. The patient was subsequently prescribed oral medications for long-term management, including Rivaroxaban (15 mg, once nightly), Bisoprolol fumarate (2.5 mg, once daily), Atorvastatin (20 mg, once nightly), and Sacubitril/Valsartan (25 mg, twice daily).

In this case, CRT-D was programmed in LV-only mode via LBBAP, with VV delay set to 0 ms. This ensured that observed improvements were attributable to LBBAP rather than biventricular fusion.

## Follow-up and outcomes

The patient consistently attended regular follow-ups, and there were no modifications or interruptions to the treatment regimen throughout the process. After six months of treatment, the patient did not experience an elevated burden of heart failure. the New York Heart Association (NYHA) functional class of the patient demonstrated an improvement, progressing from Class II to Class I. A significant improvement was observed in his left ventricular ejection fraction (LVEF), increasing from 32% to 47%, reflecting a remarkable absolute increase of 15%. Positive changes in cardiac structural parameters were documented and are detailed in Table 1.

**Table 1:** Changes of cardiac parameters at baseline and during follow-up.

Category	Parameter	Baseline	Follow-up		
			2 weeks	3 months	6 months
Structure and systolic function	LV end-diastolic diameter (LVEDD) (mm)	62	61	56	56
	LV end-systolic diameter (LVESD) (mm)	52	48	45	43
	LV end-diastolic volume (LVEDV) (mm)	195	187	155	156
	RV anteroposterior diameter (mm)	23	23	20	20
	left atrial diameter (LAD) (mm)	37	36	35	36
	mitral regurgitation	moderate	mild	mild	mild
	LVEF (%)	32	36	42	47
Electrophysiologic parameters	Electric axis (°)	−41	−6	16	17
	QRSd (ms)	168	106	104	106
	QTc (ms)	485	458	430	462
Synchronization (22)	IVMD (ms) ≥40 ms	32	28	46	46
	SPWMD (ms) ≥130 ms	123	123	137	137
	Ts-SD (ms) ≥33 ms	87	81	14	11
	LV filling time to cardiac cycle length ratio (%) >40%	53	65	58	53

We proceeded separate assessments of interventricular, and intraventricular dyssynchrony using echocardiography in the patient. In comparison to the baseline, we observed a prolonged interventricular mechanical delay (IVMD) at the 3-month and 6-month follow-up. The intraventricular radial dyssynchrony, evaluated with septal-to-posterior wall-motion delay (SPWMD), extended to over 130 ms at the 3-month follow-up. However, intraventricular longitudinal dyssynchrony, measured with the mechanical dyssynchrony index (Ts-SD), indicated a significant improvement in left ventricular longitudinal synchronization. The index decreased from 87 ms to 11 ms.

**Figure 3:**
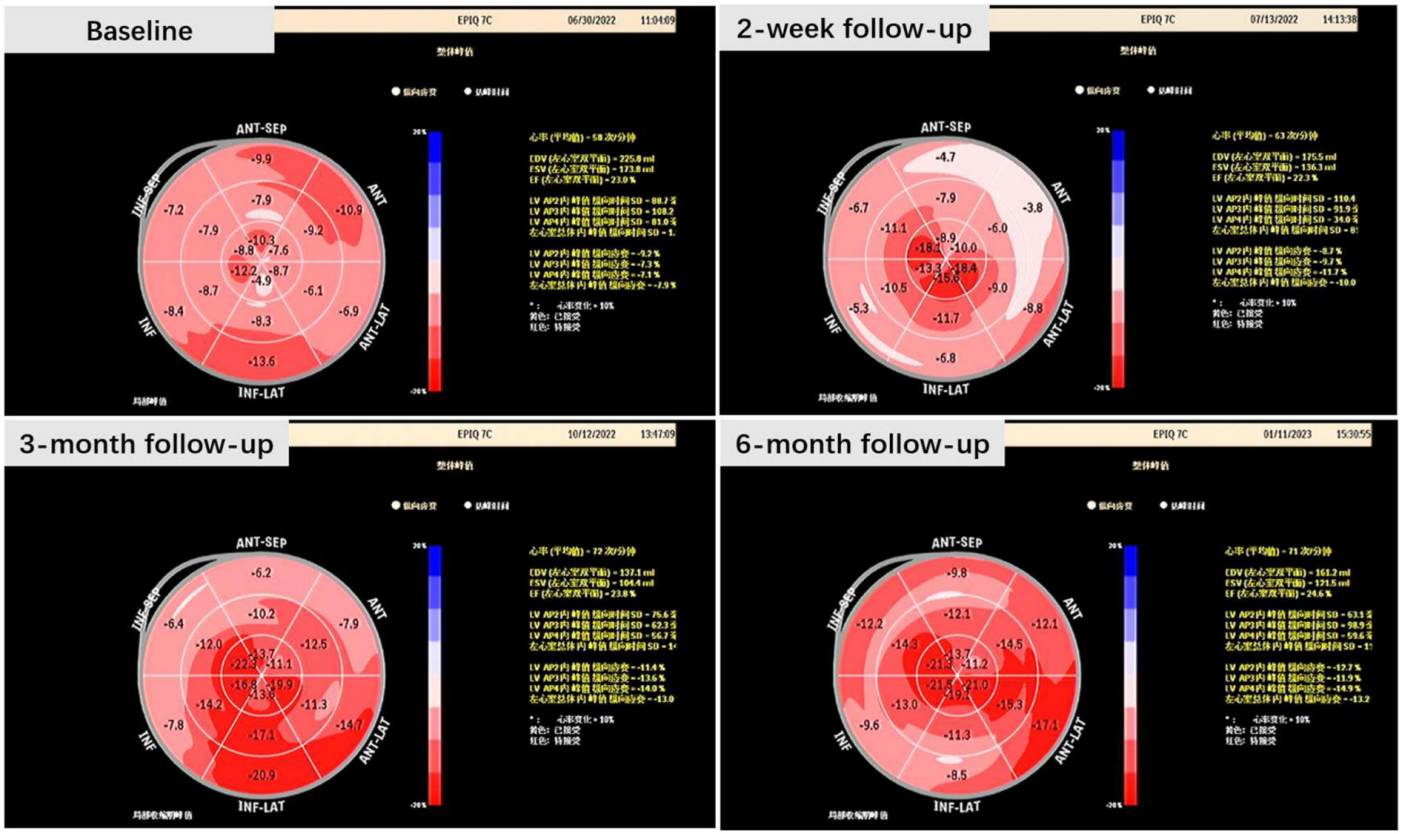
Bullseye plot at baseline and during follow-up.

The two-dimensional speckle tracking imaging generated a longitudinal strain bullseye plot, revealing an enhanced global longitudinal strain (GLS). This observation suggests a more robust contraction of the left ventricular cardiac muscle (Figure 3).

A normalized QRS duration and diminished left axis deviation was as well reported. Parameters related to the CRTD were documented both at the time of implantation and during follow-up (Table 2). There were no significant findings except for a gradual increase in the amplitude of the left bundle branch pacing electrode, progressing from 0.5 V to 0.75 V. Notably, no events, including atrial or ventricular tachycardia, or atrial fibrillation, were recorded during the follow-up period.

No procedure-related complications were recorded, such as right bundle branch (RBB) injury, significant increases in pacing threshold, dislodgement, infection, embolism, perforation, or pericardial effusion. There were no episodes of atrial fibrillation (AF) or sustained ventricular tachycardia that necessitated anti-tachycardia pacing (ATP) or shock.

## Discussion

Conduction system pacing (CSP), including His bundle pacing (HBP) and left bundle branch area pacing, has recently emerged as a promising alternative to conventional CRT (1, 2). By directly engaging the specialized conduction system, LBBAP provides more physiological ventricular activation, shortens QRS duration, and improves synchrony (3, 4). Compared with HBP, LBBAP offers a larger target area, lower and more stable thresholds, and improved lead stability. Recent studies and meta-analyses have demonstrated favorable clinical and echocardiographic outcomes, suggesting that LBBAP may represent an effective and safe alternative to conventional CRT in selected patients with heart failure and LBBB (5). Against this background, we report the present case to illustrate the potential value of LBBAP in CRT-eligible patients (6).

### Differential diagnosis

The patient presented with acute heart failure and atrial fibrillation, with further examination revealing a dilated left ventricle with systolic dysfunction and coronary artery stenosis. While coronary artery disease (CAD) is frequently identified as the most common cause of new-onset heart failure or reduced left ventricular function, in this patient, there is insufficient evidence to support the assertion that CAD led to ischemia and subsequently caused a change in heart function (7, 8).

A chronic asymptomatic LBBB made up all his past history, which could be confirmed with a partial or complete recovery of LV function after restoration of normal conduction (9–11). According to previous studies, Left Bundle Branch Block–induced Cardiomyopathy was defined by the presence of: 1) history of LBBB for more than 1 year; 2) LVEF >50% at the time of diagnosis of LBBB; 3) progressive decline in LVEF to <40% and development of New York Heart Association functional class II to IV; 4) no other identifiable cause for cardiomyopathy; and 5) echocardiographic evidence of dyssynchrony (inter-ventricular mechanical delay >40 ms; aortic preejection delay of >140 ms; septal to lateral wall delay of >65 ms) (1).

In summary, based on the criteria outlined, the diagnosis of Left Bundle Branch Block-induced Cardiomyopathy is applicable to this case, which, as reported, could be reversed with cardiac resynchronization therapy (12).

### Efficacy

Although guideline-directed medical therapy (GDMT) remains the cornerstone of heart failure management, our patient exhibited symptomatic hypotension and did not tolerate further uptitration prior to CRT-D implantation.

Moreover, recent evidence supports the notion that early correction of LBBB may confer superior clinical outcomes compared to delayed intervention. The NEOLITH study (13) demonstrated that GDMT alone did not significantly improve LVEF in patients with new-onset LBBB-associated cardiomyopathy after 3 months. Notably, a large proportion of these patients remained CRT candidates, and approximately 35% became super-responders once resynchronization therapy was initiated.

Similarly, the NEOLITH II study (14) showed that patients who received biventricular pacing (BVP) within 9 months of LBBB-associated cardiomyopathy diagnosis experienced more favorable cardiac remodeling than those treated later. These findings suggest that postponing device implantation may result in a missed therapeutic window for halting disease progression and reversing myocardial dysfunction. Early intervention with conduction system pacing—such as HBP or LBBAP—may help normalize electrical activation and promote myocardial recovery.

Studies on Cardiac Resynchronization Therapy-Defibrillator implantation have reported that, when compared with conventional pacing sites, LBBAP exhibits a high success rate in implantation. LBBAP has proven effective in correcting LBBB while simultaneously improving left ventricular structure and function, all with a low and stable pacing threshold (15, 16). Despite these positive findings, current guidelines (3, 4) do not provide specific recommendations for the use of LBBAP, emphasizing the need for additional evidence regarding its safety and efficacy.

In our case, the paced QRS complex demonstrated a V6 RWPT of 93 ms and a V6–V1 interpeak interval of 10 ms, which did not fully meet the strict definition of selective left bundle branch pacing. Nevertheless, recent findings provide further context for these observations. Shen et al. (17) reported that RWPT can vary dynamically depending on pacing output and fascicular involvement, while Ponnusamy et al. (18) emphasized that non-selective capture can still restore near-physiological ventricular activation and confer meaningful clinical benefit. These observations align with the patient's marked QRS narrowing and echocardiographic improvement, supporting the clinical efficacy of LBBAP in this case.

Responders and super-responders to Cardiac Resynchronization Therapy are typically defined by an absolute change in left ventricular ejection fraction (LVEF) of >5% and >15%, respectively, at the 6-month follow-up (19–21). In this case, the patient achieved a remarkable 15% absolute increase in LVEF, reaching the borderline of super-response. This suggests an excellent response to Left Bundle Branch Area Pacing with Cardiac Resynchronization Therapy-Defibrillator in patients with LBBB-induced cardiomyopathy.

### LBBAP increase conduction velocity of LBB

The preoperative electrocardiogram indicated a left-axis deviation, while the postoperative electrocardiogram immediately showed a restoration to a normal axis. There are two possible reasons for the change in axis: 1. Alteration in the sequence of ventricular excitation in the conduction system, such as in this case where the left anterior branch conduction speed increased, correcting the relative left anterior branch block; 2. Gradual restoration of cardiac structure, which takes some time. Intraventricular longitudinal mechanical delay was significantly reduced, with the longest delay consistently observed at the anterior wall. This suggests that LBBAP did not modify the sequence of depolarization of the left bundle branch; instead, it might have accelerated the conduction of the left bundle branch, especially the left anterior fascicle, facilitated by the pacing electrode providing a higher conduction velocity.

**Figure 4:**
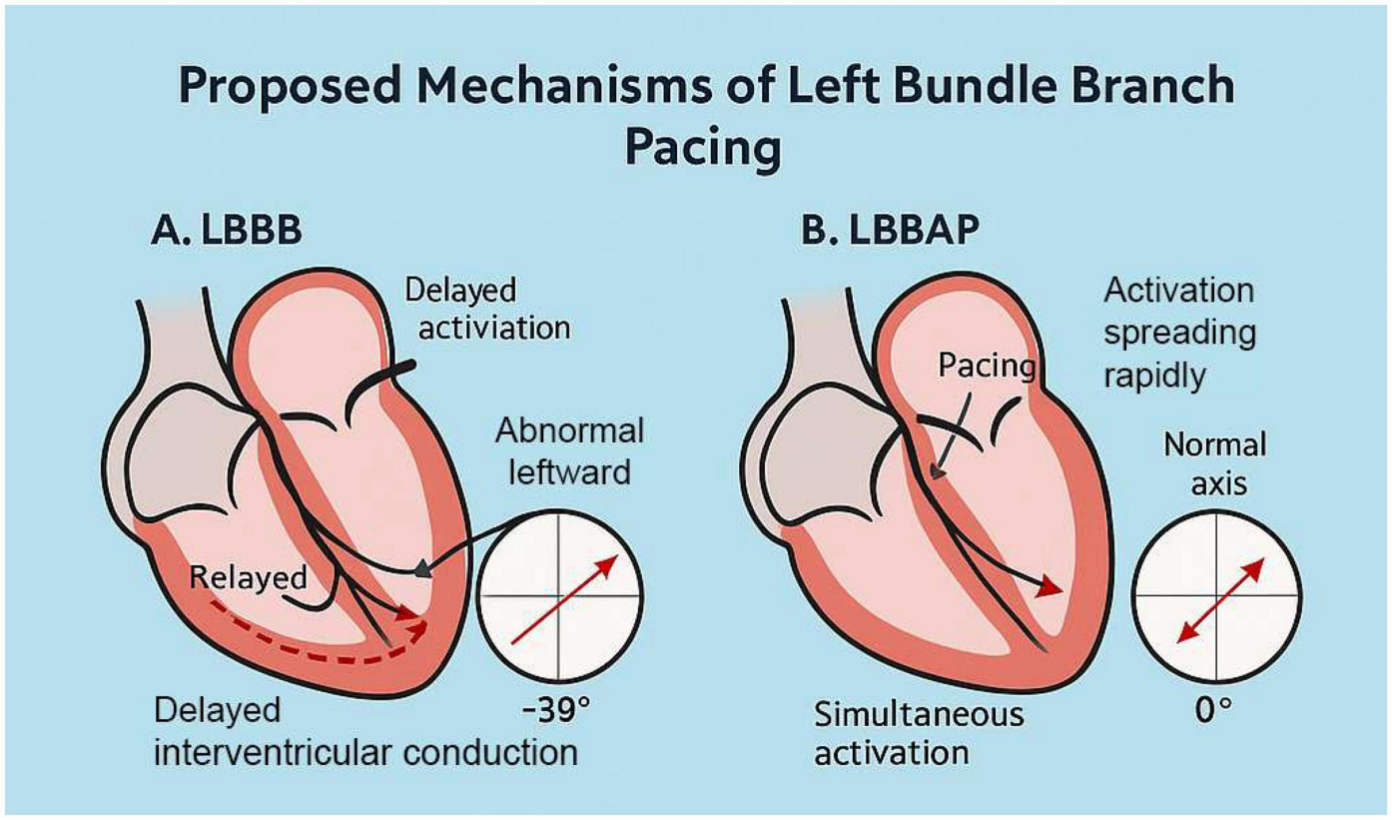
Comparison of conduction pattern and ECG features in LBBB vs LBBAP.

As shown in Figure 4, LBBAP preserves physiological ventricular activation and restores frontal axis orientation by effectively bypassing the site of conduction block. The resolution of left anterior fascicular block (LAFB) was indicated by normalization of the QRS axis following LBBAP. However, this finding may also be influenced by fusion pacing, interindividual variation in septal anatomy, or conduction heterogeneity. We acknowledge that surface ECG alone may not be sufficient to distinguish between true improvements in conduction velocity and axis normalization secondary to altered activation pathways. Further validation with invasive electrophysiological mapping would be necessary to confirm this hypothesis.

Regarding intraventricular dyssynchrony, we measured both radial and longitudinal dyssynchrony of the left ventricle. Septal-to-posterior wall-motion delay (SPWMD) revealed radial dyssynchrony, while the mechanical dyssynchrony index (Ts-SD) disclosed longitudinal dyssynchrony.

There was a notable decrease in Ts-SD, indicating a reduced standard deviation of the time to peak myocardial velocity (Ts) across the 18 left ventricular (LV) segments. This implies a shortened time interval or a higher velocity for longitudinal conduction within the left ventricle. However, an extended interval between the maximum contraction of the septum and the left ventricular posterior wall, as revealed by SPWMD, was observed. This could potentially be explained by an enhanced conduction along the septal area rather than delayed activation of the posterior wall. In other words, the improvement in the longitudinal conduction of the LV led to an observed impairment of radial conduction.

The activation sequence of the left ventricle remained unchanged during the follow-up, with the longest delay consistently observed at the basal segment of the anterior and inferior lateral wall. This further supports the indication that LBBAP did not modify the activation sequence of the left ventricle.

The hypothesis that LBBAP improves left bundle conduction velocity is based on indirect evidence from ECG axis normalization and improved Ts-SD values. However, direct assessment of conduction velocity (e.g., via intracardiac electrograms or His-Purkinje mapping) was not performed in this case. We acknowledge this as a limitation.

Therefore, since Left Bundle Branch Area Pacing with Cardiac Resynchronization Therapy-Defibrillator significantly improved the structure and function of the left ventricle, we can conclude that the cardiomyopathy induced by LBBB is not primarily due to an abnormal activation sequence among different segments of the left ventricle. Instead, it is attributed to the prolonged conduction time of the left bundle branch. With the improvement in conduction velocity of the left bundle branch, the patient's heart function and structure were restored.

## Conclusion

We presented the case of a patient with left bundle branch block-induced cardiomyopathy and heart failure with reduced ejection fraction. The patient underwent Left Bundle Branch Area Pacing with Cardiac Resynchronization Therapy-Defibrillator and achieved a remarkable absolute 15% improvement in left ventricular ejection fraction at the 6-month follow-up. Importantly, there was no alteration to the left ventricular activation sequence; instead, an improved conduction velocity of the left bundle branch was observed. This suggests that LBBAP-CRTD holds promise as a treatment modality for patients with LBBB-induced cardiomyopathy, demonstrating outstanding safety and efficacy.

This is a single case report, and the findings should be interpreted with caution. Further prospective studies with larger sample sizes are needed to confirm whether the observed conduction velocity improvement represents a consistent effect of LBBAP.

## Data Availability

The original contributions presented in the study are included in the article/Supplementary Material, further inquiries can be directed to the corresponding author.
