# Supplementary material for: Case Report: Left bundle branch area pacing in cardiac resynchronization therapy increases conduction velocity
Source: Front Cardiovasc Med. 2025 Oct 3;12:1620302. doi: 10.3389/fcvm.2025.1620302 (PMC12531258; doi:10.3389/fcvm.2025.1620302)

Supplementary Figure 1
V6 RWPT 93ms and V6–V1 interpeak interval 10ms

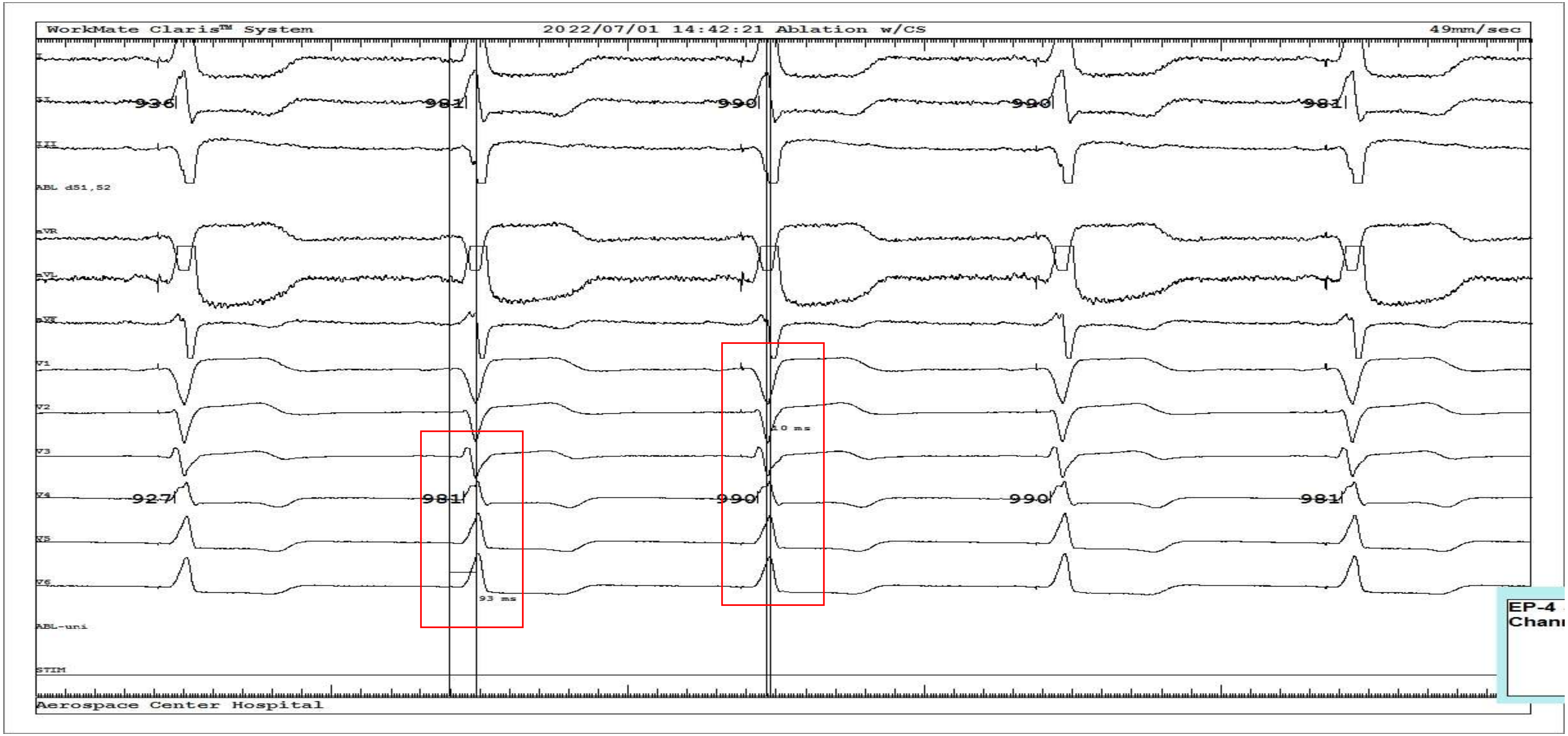

Supplementary Figure 2 High power output in testing

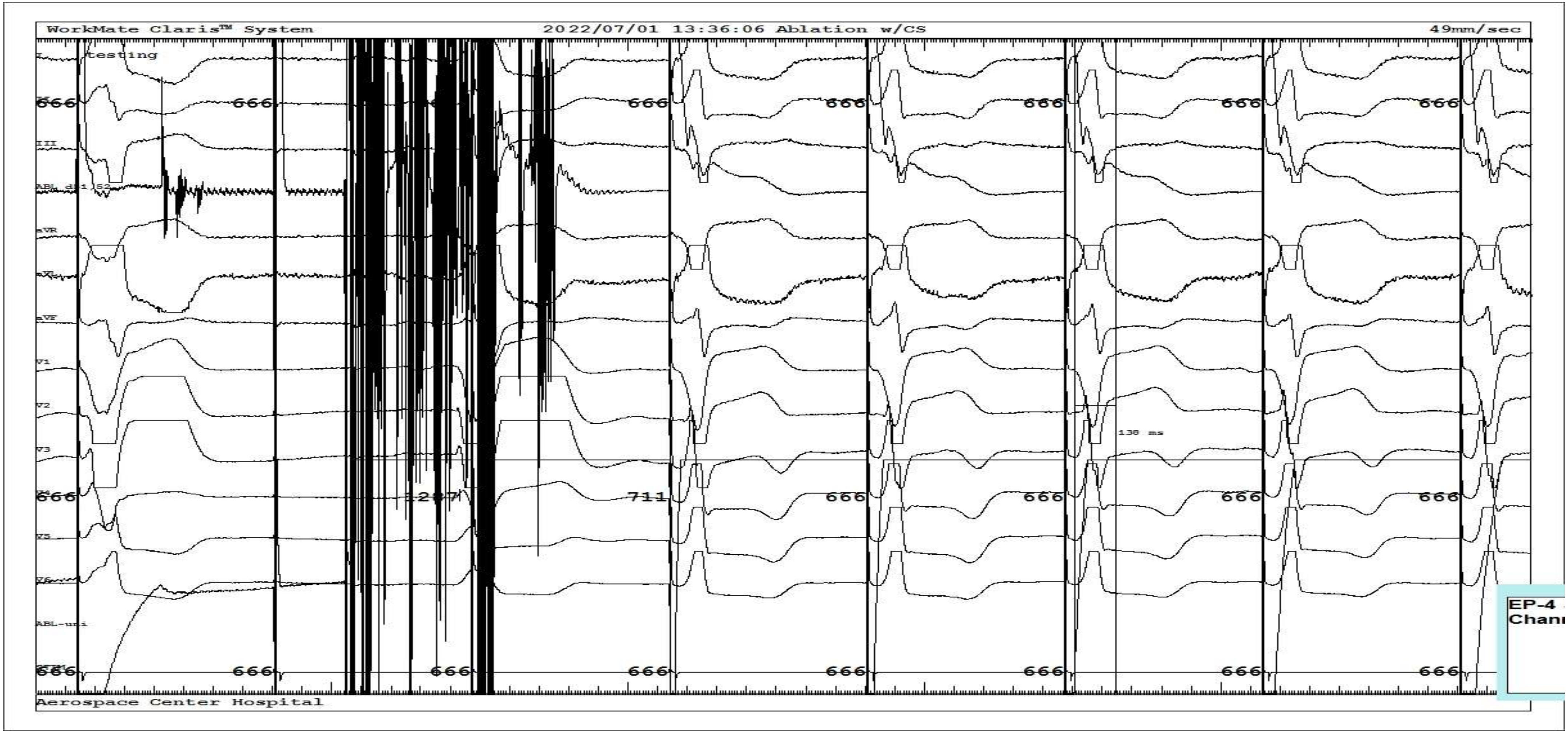

Supplementary Figure 3 Low power output in testing

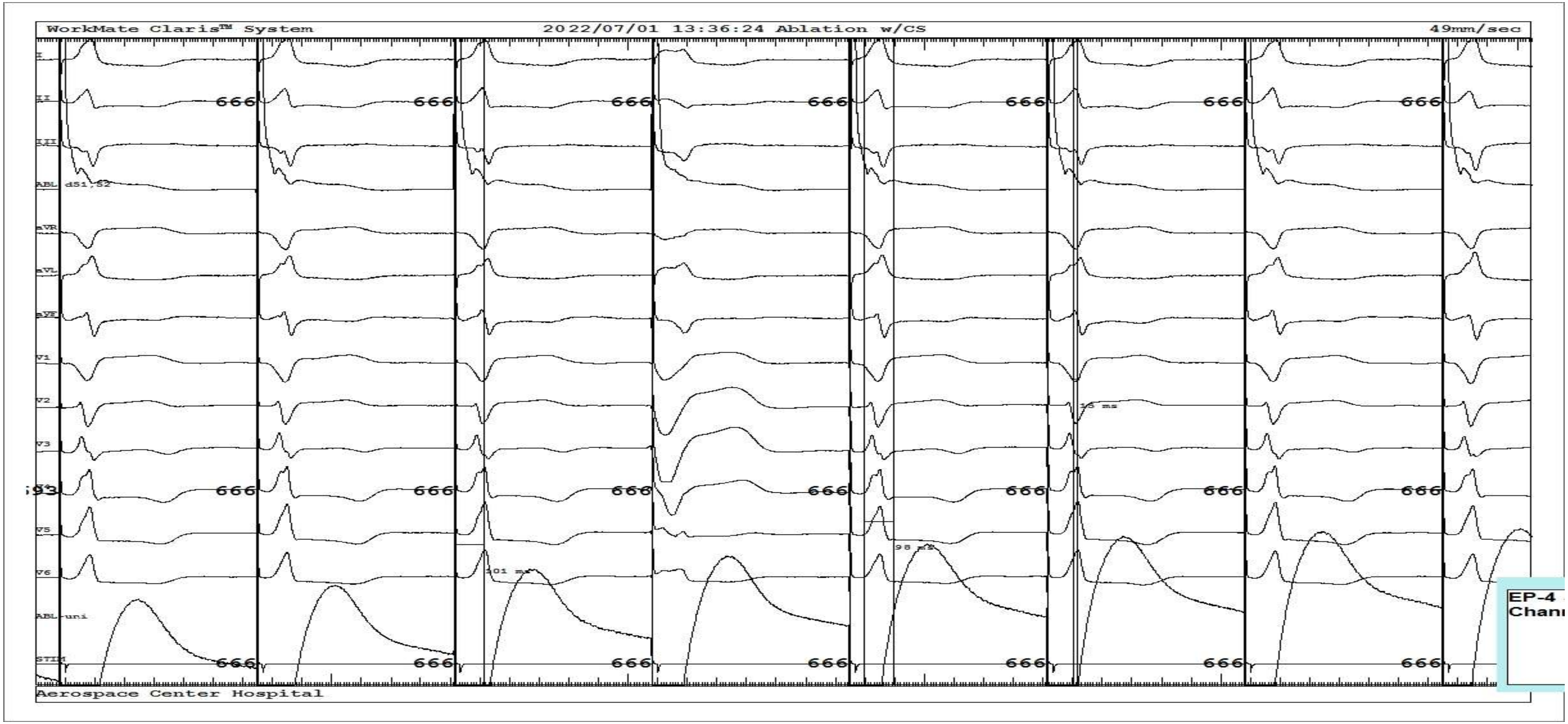

Supplement: Supplementary file 1 [file Datasheet1.pdf]
